# Hook Wire Localization Procedure and Early Detection of Breast Cancer - Our Experience

**DOI:** 10.3889/oamjms.2015.055

**Published:** 2015-05-19

**Authors:** Maja Jakimovska Dimitrovska, Nadica Mitreska, Menka Lazareska, Elizabeta Stojovska Jovanovska, Ace Dodevski, Aleksandar Stojkoski

**Affiliations:** 1 *University Clinic of Radiology, Medical Faculty, Ss. Cyril and Methodius University of Skopje, Skopje, Republic of Macedonia*; 2 *Institute of Anatomy, Medical Faculty, Ss. Cyril and Methodius University of Skopje, Skopje, Republic of Macedonia*

**Keywords:** mammography, BI-RADS classification, wire localization, breast cancer

## Abstract

**AIM:**

The purpose of this study is to describe our experience with needle localization technique in diagnosing small breast cancers.

**MATERIAL AND METHODS:**

This retrospective study included a hundred and twenty patients’ with impalpable breast lesions and they underwent wire localization. All patients had mammography, ultrasound exam and pathohystological results. We use Mammomat Inspiration Siemens digital unit for diagnosing mammography, machine - Lorad Affinity with fenestrated compressive pad for wire localization and ultrasound machine Acuson X300 with linear array probe 10 MhZ. We use two types of wire: Bard hook wire and Kopans breast lesion localization needle, Cook. Comparative radiologic and pathologic data were collected and analyzed.

**RESULTS:**

In 120 asymptomatic women, 68 malignancies and 52 benign findings were detected with mammography and ultrasound. The mean age for patients with malignancy was 58.6 years. According BI-RADS classification for mammography the distribution is our group was: BI-RADS 3 was presented in 6 (8.82%) patients, BI-RADS 4 was presented in 56 (82.35%) patients and BI-RADS 5 was present in 6 (8.82%) of the patients. Most wire localizations were performed under mammographic guidance in 58 from 68 patients with malignant lesions (85.29%) and with ultrasound in 10 (14.7%). According the mammographic findings patients with mass on mammograms were 29 (42.65%), mass with calcifications 9 (13.23%), calcifications 20 (29.41%) and architectural distortions or asymmetry 10 (14.71%).

**CONCLUSION:**

Wire localization is a well established technique for the management of impalpable breast lesions.

## Introduction

Breast cancer is the most common form of malignancy occurring in women [1]. Mammography provides the opportunity to detect breast cancer at early stage, when it is nonpalpable and likelihood for cure is great [2-4].

Wire localization of nonpalpable mammographically detected breast lesion is a well established technique, the importance of wich has grown recent years with the advent of mammographic breast screening programmes and an increasing use of mammography in the investigation of symptomatic breast disease [5].

Mammographic detection of nonpaplable breast cancer permits earlier diagnosis and almost certainly reduces mortality from the disease [3, 4, 6, 7].

The key to successful management of these nonpalpable lesions is accurate localization, which is essential for achieving complete surgical excision with optimal cosmetics and minimal morbidity. Imaging guidance is most commonly performed with mammography or sonography and less often with MRI or CT [8]. Patients with breast cancer diagnosed in earlier stage are less likely to need extensive treatment with potentional for harmfull side effects and have a reduce risk of recurrence [9].

The aim of our study is to describe our experience with needle localization technique which we believe to be a safe and accurate method for gaining access to small and highly curable breast cancers.

## Material and Methods

From November 2009 till November 2014, we performed 120 wire localizations in the University Clinic of Radiology in Skopje, Republic of Macedonia. All patients had operative treatment at the University Clinic for thoracovascular surgery. The cytological analysis was done at the department of cytology and histology at the University Clinic of radiotherapy and oncology. Pathohystological analysis was performed in department of cytology and histology at the University Clinic of radiotherapy and oncology and in the Institute of pathology, Medical Faculty, Skopje, R. Macedonia. We retrospective analyzed 68 patients with histology proven breast cancer, mean age 58.66 years. The charts of all patients undergoing wire localization were reviewed for following: age, size of the lesion, quadrant of breast where the lesion appeared, method of localization, degree of suspicion in mammographic findings, presence of microcalcifications, number of lymph nodes involved, and final pathologic diagnosis. The mammograms were assessed using the Breast Imaging Reporting and Data system (BI-RADS) classifications: BI-RADS 0 need additional Imaging Evaluation or prior mammograms; BI-RADS 1 negative. There is nothing to comment on; BI-RADS 2 Benign finding; BI-RADS 3 Probably benign finding (<2% malignant); BI-RADS 4 Suspicious abnormality (2-95% malignant); BI-RADS 5 Highly suggestive of malignancy (>95% malignant); BI-RADS 6 Known biopsy-proven malignancy [10].

In our study we include BI-RADS 3, 4 and 5. All patients underwent diagnostic mammography at our institution. The mammographic images were reviewed to confirm the absence or presence of a mass, mass with calcifications, architectural distortion and microcalcifications.

The patohistological results were describe according TNM classification of malignant breast tumors.

Tumors are often graded based on a scale of one to three indicating how aggressive the cancerous cells are:

Low grade (1) - Well-diffentiated;

Intermediate grade (2) - Moderately differentiated;

High grade (3) - Poorly differentiated.

The procedure begins in the Breast Imaging Department. The study was performed using imaging equipment by Mammomat Inspiration Siemens digital unit for diagnosing mammography; machine - Lorad Affinity with fenestrated compressive pad for wire localization and ultrasound machine Acuson X300 with linear array probe 10 MhZ. We use two types of wires: Bard hook wire and Kopans breast lesion localization needle, Cook.

Patients were in supine position with examined side by a small pillow and ipsilateral arm raised above head during the ultrasound wire localization. Mammography wire localization was performed in sitting position. The technique involves mammography usually requires the upright mammographic attachment on a normal mammographic unit. Localization was performed under local anesthesia with lidocain. Before the localization a grid or holey plate was used to accurate the position of needle placement in the X and Y planes. The depth was calculated from the lateromedial projection. The position was then checked according to the superimposition of target, hub and shaft of the needle and the required depth was verified on the orthogonal view. Needle is inserted into the breast, directed towards the lesion and taped in place.

After surgical removal of the lesion, specimen radiography was performed to ensure that the lesion was adequately excised. The average time required for complete procedure was about 25-30 minutes. Patients discomfort was minimal and no major complications occurred. All patients provided written informed consent before the procedure.

Good accuracy of such localizations is required to ensure correct and adequate removal of the lesion and to minimize the degrees of cosmetic disfigurements.

Statistical analysis of data obtained during the study was done with statistical program SPSS 13.0 for Windows. Results of the study were presented with descriptive statistics and with distribution of frequency. The valid of the diagnostic test mammography is testing with using pathophysiologic results as a golden standard.

## Results

The average age of patients with malignant disease was 58.66 ± 7.6 years.

Familiar data for breast cancer had 15 patients (22.06%).

In this study we analyzed 120 patients who were preoperative wire localized and 68 (56.66%) patients were with breast cancer and in 52 (43.33%) patients were with benign findings.

In left breast were 38 (55.88%) and in right breast 30 (44.12%) were malignant lesions. The lesion localization by quadrants was dominant on superior lateral quadrant in 53 (77.94%), superior medial quadrant 4 (5.88%), inferior medial quadrant in 5 (7.35%), inferior lateral quadrant in 5 (7.35%) and retromammillar space in 1 (1.47%).

The lesions are visible by ultrasound in 42 patients (61.76%) and 26 (38.23%) lesions were not visible by ultrasound. All lesions were detected by mammography.

We analyzed the shape of the masses, the results with the mammography examination showed that the mass was irregular in 28 (41.18%), round in 15 (10.29%) and oval in 10 (14.71%). On ultrasound examination the margins are not circumscribed in 40 (58.82%) and they were indistinct and speculated.

The lesions visible by ultrasound were classified in three groups according to the dimensions. In the first group we have 7 (10.29%) patients and the diameter of the lesion was ≤ 5 mm. In the second group we found 24 (35.29%) patients with the diameter of the lesion in range from 6 to 10 mm. In the third group we found 10 (14.71%) patients with the diameter of the lesion in range from 11 to 20 mm.

**Figure 1:**
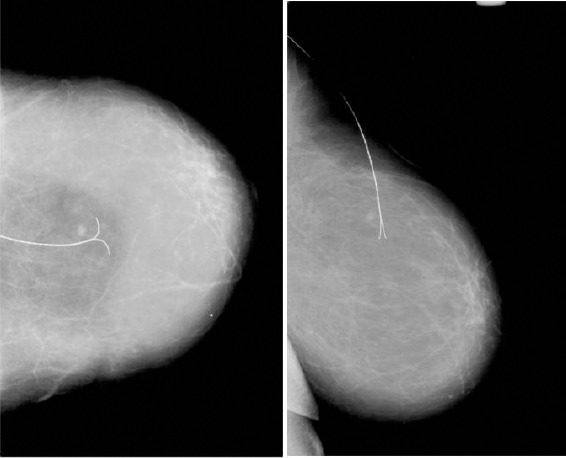
*Wire localized small impalpable mass in the breast on CC and LM projection*.

Our study included only patients who had needle localization but didn’t include all patients underwent mammography in our department. Most wire localization was performed under mammographic guidance in 58 from 68 patients with malignant lesions (85.29%) and with ultrasound in 10 (14.7%). According the mammographic features all patients were divided in 4 groups, the results are presented in Table 1.

**Table 1:** Classification of mammographic findings

Groups	N (%)
Mass	29 (42.65%)
Mass with calcifications	9 (13.23%)
Microcalcifications	20 (29.41%)
Arhitecture distorsion and asymmetry	10 (14.71%)

According BI-RADS classification for mammography the distribution is our group was: BI-RADS 3 was presented in 6 (8.82%) patients, BI-RADS 4 was presented in 56 (82.35%) patients and BI-RADS 5 was present in 6 (8.82%) of the patients.

**Table 2:** Distribution of the patients according to the BI-RADS classification

BI-RADS	1	2	3	4	5
Mammography			6 (8.82%)	56 (82.35%)	6 (8.82%)
Ultrasound	24 (35.29%)	1 (1.47%)	4 (5.88%)	38 (55.88%)	1 (1.47%)

The cytological analysis was performed in 50 patients from 68 in the group at the department of cytology and histology at the University Clinic of radiotherapy and oncology. According to the cytological analysis the patients were distributed in the following groups: I group 37 (54.41%), III group 7 patients (10.29%), IV group 2 (2.24%) and V group 4 (5.88%) of the patients.

In this study we didn’t performed core biopsy in any patients.

The pathohystological results in our study are presented in Table 3.

**Table 3:** Pathohystological analysis of the patients

Pathohystological results	N	%
DCIS	7	10.29
Invasive ductal carcinoma	44	64.70
Lobular in situ carcinoma	3	4.41
Invasive lobular carcinoma	6	8.82
Others	8	11.76

According to the grade of the tumor grow the results were G1 stage in 17 (25%) patients, G2 stage in 41 (60.29%) and G3 stage in 10 (14.71%) patients.

**Figure 2:**
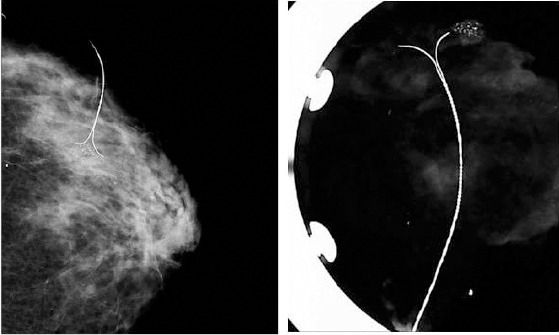
*Wire localized microcalcifications in ductal carcinoma in situ and postoperative radiogram of the specimen*.

## Discussion

Preoperative placement of a hook wire into nonpalpable lesions under imaging guidance is commonly perform for patients with suspective findings on mammograms and/or ultrasound for breast cancer. We found that this technique is extremely useful for small lesions and microcalcifications. Although our group of proven cancers is small, it can be representative and comparable with those in literature.

In our study the mean age of the patients is 58.66 years old.

Sickles reported mean age 57 years old [11]. Basset et al. in study of 207 patietns with impalpable lesion inform about mean age of 59 years [12].

The data from the previous published studies are in accordance with our study. Our patients are older, which is not a trend in incidence of breast cancer, but it is also a technical factor; that smaller lesions are more easily identified in atrophic breast than in the younger, denser breast tissue.

The lesions are visible in 61.76% by ultrasound and 38.23% lesions are not visible by ultrasound.

Of 120 cases, 68(56.6%) were malignant and 52(43.33%) were benign. Ductal carcinoma insitu and microinvasivum are in 17.64%.

In 308 cases, Shin et al. reported malignancy in 47% and benign lesions 53%. Ductal carcinoma insitu including microinvasivum are in 108(35%) [13]

Distribution of types of cancer among our group are: invasive ductal carcinoma in 51 cases (75%), invasive lobular carcinoma 9(13.23%), tubular carcinoma in 6(8.82%) and others 2(2.54%).

The study of 72 impalpable cancers were 39(54%) invasive ductal, 23(32%) noninvasive ductal, lobular carcinoma 4 (6%), tubular 1(1%), colloid 5(7%) [12].

Other study of 151 cases reported 83(55%) invasive ductal carcinoma, insitu duct cancer 40(26.4%), microinvasive duct cancer 9(6%), invasive lobular 5(3.3%), lobular neoplasia 13(8.6%) [14]

The incidence of malignancy in our study is little higher because our center I the only public center in the country where the procedure is performing. Also we use magnification mammography in diagnosting small breast lesions instead of alternative approach of periodic mammographic follow up.

In our study cancer representing with microcalcifications without mass in 29.41%, mass is in 42.65%, mass with calcifications in 13.23% and architecture distorsion and asymmetry in 14.71%.

Microcalcifications are estimated to be associated with malignancy in approximately 25% to 35% of cases [15]. Calcifications associated with mass have been reported to be particular ominous mammographic findings as they are associated with carcinoma in 50% of cases [15] and 83% by Schwartz et al. [14].

Architectural distortion is the third most common mammographic appearance of non palpable cancer, representing 6% of abnormalities detected on screening mammography [16].

Gardenosa and others showed distribution of 543 lesions: mass 224(41%), calcifications 254(47%), mass and calcifications 41(7.55%), architectural distortion 24(4%) [17].

Our results are comparable with these ranges in literature.

In our study predominant is ductal cancer in 51(75%) patients.

In the reported series involving needle localization technique, the predominant pathologic cancer type in the literature is ductal [9].

In our group microcalcifications associated with malignancy were in 29.41%(20/68).

Microcalcifications are estimated to be associated with malignancy in approximately 25% to 35% of cases [14].

In our study dominate intermediate grade 2 tumor grow in 41/68(60.29%), which means moderately differentiated.

Gajdos and others reported cancers with intermediate grade in different groups but in all of them dominate G 2 grade [18]. Our results are according the literature.

False negative FNAC results have been associated with certain features (e.g. small tumor size, low ceilularity and special type histology) wich may be commonly observed with impalpable mamographically detect breast lesions then with palpable abnormalities. We rarely recomend biopsy in an inpalpable asymetric density that shows microcalcifications.

In our study 97% (66/68) of wire localized lesions, the wire crosses the lesion.

In hookwire localization of breast lesions, the cannula is typically advanced through the lesion and the hook-wire deployed so that the thickened portion of wire is located across the lesion [19].

Also in 53/68 (77.94%) patients had localization in superolateral quadrants.

Distribution of BI-RADS classification for mammography among our group shows that dominates BI-RADS 4 and it is 82.33%.

Theoretically, BI-RADS 4 are indeterminate lesions and malignancy rates can range from 2% to 95% [20].

Our results indicate that hook wire localization of small lesions and microcalcifications permits the surgeon consistent excision of mammographic lesion safely.

Careful comunication between radiologist and surgeon appears however to be of great importance. We found it particulary helpful for radiologist to relay verbally to the surgeon.

Also Schwartz et al. emphasis close cooperation between surgeon, radiologist, and pathologist insures that the suspicious area(s) are removed in their entirety with the sacrifice of the minimal amount of contiguous normal breast tissue [14].

Wire localization is a procedure that uses a mammogram or ultrasound to locate and identify breast lesion. Using the mammogram as a guide the radiologist locates the area of concern. It is a good tool for marking impalpable and small lesions.

Diagnostic mammography is most helpful in deciding a nonpalpable breast lesion should be wire localized. When an abnormality is detected, clinical evaluation and thorough radiologic work up are needed to determine the suspicious for cancer.

Wire localization is a well established technique for the diagnosis of impalpable breast lesions.
